# Psychiatric Comorbidities and the Risk of Suicide in Obsessive-Compulsive and Body Dysmorphic Disorder

**DOI:** 10.7759/cureus.9805

**Published:** 2020-08-17

**Authors:** Noha Eskander, Therese Limbana, Farah Khan

**Affiliations:** 1 Psychiatry, California Institute of Behavioral Neurosciences & Psychology, Fairfield, USA

**Keywords:** public psychiatry

## Abstract

Obsessive-compulsive disorder (OCD) is a chronic psychiatric disorder that is characterized by obsessions and compulsions. Obsessions are uncontrollable distressful thoughts. Compulsions are recurrent behaviors or thoughts performed in an attempt to decrease the anxiety of the obsessions. Body dysmorphic disorder (BDD) is a mental disorder characterized by a distressful preoccupation with a perceived defect in appearance. The perceived flaw in appearance is minimal or unnoticed by others. BDD was considered an anxiety disorder in the Diagnostic and Statistical Manual of Mental Disorders, fourth edition (DSM-IV). In the DSM-V, it was added to the obsessive-compulsive and related disorders category. The objective of this literature review was to explore the psychiatric comorbidities and the risk of suicide associated with OCD and BDD. Our study results showed OCD and BDD share common genetic and environmental risk factors, clinical features, and sociodemographic profiles. Both OCD and BDD are related disorders that commonly coexist. The suicide risk in OCD is increased as the intensity of the obsessions, trait perfectionism, and alexithymia increases. The suicide risk in BDD is increased by the presence of other disorders such as substance use disorder, major depressive disorder, eating and personality disorders. People with comorbid OCD-BDD have high morbidity, a decrease in insight and poor psychosocial functions. They have higher rates of anxiety, schizotypal features, and suicidal ideation compared to those with BDD or OCD alone.

## Introduction and background

Obsessive-compulsive disorder (OCD) is a chronic mental disorder. It is characterized by obsessions and compulsions. Obsessions are uncontrollable recurring and distressful thoughts. Compulsions are repetitive behaviors which the person feels the urge to perform in an attempt to decrease the anxiety of the obsessions. The estimated prevalence of OCD among adults in the United States for a 12-month period is 1.2% and for a lifetime is 2.3%. The mean age of OCD onset is 19.5 years [1]. There is a significant gender difference in the OCD age of onset. Males usually start developing symptoms before the age of 10 compared to females who usually start to develop symptoms after the age of 10. Most individuals with OCD spend a mean of 8.9 years of their life with the disorder [1].

Body dysmorphic disorder (BDD) is also known as dysmorphophobia. BDD was considered an anxiety disorder in the Diagnostic and Statistical Manual of Mental Disorders, fourth edition (DSM-IV). But in the DSM-V was added to the obsessive-compulsive and related disorders category. BDD is a psychiatric disorder characterized by an excessive, persistent, and distressful preoccupation with a perceived defect in appearance. These perceived defects are slight and are unnoticed by others. People with BDD usually have poor insight and are preoccupied with a perceived physical defect which causes them to check on it repeatedly. This leads to an impairment in psychosocial functioning, depression, and an increase in suicide risk [2].

The prevalence of BDD in the adult community is 1.9%, 7.4% in psychiatric inpatients, 13.2% in general cosmetic surgery, 20.1% in rhinoplastic surgery, and 9.2% in cosmetic outpatient clinics [3]. BDD is an underdiagnosed disorder. Many people suffering from BDD do not seek psychiatric advice, instead, they are usually diagnosed in cosmetic clinics and other settings because they do not recognize they have a problem. Others prefer not to disclose their condition because they are afraid to be judged or because they feel embarrassed [4]. BDD is usually diagnosed in adolescence [5]. It is more common in females than males with a prevalence of 2.1% and 1.6%, respectively [2].

Historically, the risk of suicide in patients with OCD was considered low [6]. Recent studies found about 63% of individuals with OCD had suicidal thoughts and 26% had suicidal attempts [7]. The presence of comorbid disorders significantly increases suicide risk [7]. People with BDD are four times more likely to experience suicidal ideation and 2.6% more likely to attempt suicide than individuals without BDD [8].

The objective of this literature review was to explore what psychiatric comorbidities are associated with OCD and BDD and to review the risk of suicide in these two disorders. The study review also aimed to understand the risk factors, clinical differences, comorbidities, and suicide risk in comorbid OCD and BDD.

## Review

Methods and results

Data were searched on Pub Med using regular keywords “Obsessive-Compulsive Disorder”, “Body Dysmorphic Disorder”. Table 1 shows the search results of the regular keywords “Obsessive-Compulsive Disorder”, “Body Dysmorphic Disorder”.

**Table 1:** PubMed keywords search results

Keywords	Database	Date	Number of results
Obsessive-compulsive disorder	Pub Med	7/25/2020	20,423
Body dysmorphic disorder	Pub Med	7/25/2020	1,862

Results

The total number of keywords search results was 22,285. The following is a breakdown of the keywords searched and the volume of results: Obsessive-Compulsive Disorder keyword search results were 20,423. Body Dysmorphic Disorder keyword search results were 1,862.

Only research articles related to human studies that were published since 1998 in the English language were included in this study. All types of research articles were included except for books and documents. After the manual screening of each article, the relevant research studies for this literature review were selected. A total of 31 articles were selected for this study to determine the psychiatric comorbidities and the risk of suicide in obsessive-compulsive and body dysmorphic disorder.

Discussion

The results of the study showed that both OCD and BDD are associated with poor psychosocial outcomes, an increase in the associated comorbidities, and suicidal risk. Individuals with comorbid OCD and BDD are at an increased risk of depression, anxiety, and suicide.

Obsessive-Compulsive Disorder

OCD is a common mental disorder that is often under-diagnosed. Patients with OCD might wait for years before they seek medical help or receive the right diagnosis. This is due to a lack of awareness and stigma around OCD. Symptoms of OCD include obsessions such as fear of contamination, doubts about harm, unacceptable thoughts and concerns about symmetry, and their respective compulsions in response to their obsessions such as cleaning, repeatedly checking on things, mental rituals, and placing items in order [9]. About 25% of OCD cases reported obsessions that are not associated with compulsions [10]. Neuroimaging studies showed that OCD is associated with an increase in the activity in a brain circuit that involves the orbitofrontal cortex (OFC), striatum, and thalamus. These findings might reflect glutamatergic dysfunction in OFC-striatal pathways. OCD is also associated with Serotonergic dysfunction based on the efficacy of SSRI (selective serotonin reuptake inhibitors) in treating OCD [11]. Genetic studies showed OCD is a heritable disorder that runs in certain families with 45% to 65% genetic contribution [12].

Approximately 90% of individuals with OCD have other psychiatric comorbidities. The most common comorbid disorder in OCD is anxiety disorders with a prevalence of 75.8%, mood disorders with 63.3% specifically major depression disorder (MDD) with 40.7%, impulse control disorders 55.9%; and substance use disorders (SUDs) 38.6 % [7]. ADHD, tic disorders, and hypochondriasis are also common with OCD [13]. The comorbidity of OCD and depression significantly increases the risk of suicide [7]. It is believed that OCD symptoms predate depression due to the distressful and the functional impairment experienced by people with OCD. Depression is more associated with obsessions than compulsions, the higher the frequency and intensity of the obsessions, the higher the number of suicide attempts [14]. A systematic review by Albert et al. found the severity of comorbid anxiety symptoms in OCD patients is associated with a high risk of suicide [15]. Additionally, comorbid bipolar disorder (BD), cigarette smoking, and a history of abuse all predict higher suicidal risk in patients with OCD [15].

A study by Kim et al. (n = 81) found that trait perfectionism is associated with high suicidal risk [16]. A cross-sectional study (n = 548) found the increase in the suicidal continuum is associated with an increase in aggression, religious and sexual thoughts, and concerns about symmetry and order [17]. Other factors that were associated with suicide were previous suicide attempts and a family history of suicide [17]. A cohort study (n = 36,788) found that OCD patients are ten times more likely to commit suicide than the general population [6]. Women are more likely to attempt suicide than men. The most method used to commit suicide in OCD patients was self-poisoning possibly due to the availability of psychotropic drugs. Comorbid personality disorder and substance use disorders were also associated with a higher risk of suicide [6]. A case report study described a case of OCD with suicidal obsessions [10]. The study emphasized the importance to differentiate suicidal ideation from suicidal obsessions. Suicidal obsessions are stressful, repetitive, and intrusive thoughts that are highly undesirable and avoidable. Suicidal obsessions are not associated with a desire to act on them. Suicidal ideations are thoughts about self-harm with serious intent to act on them and they are not necessarily intrusive or stressful. It is important to properly investigate patients presenting with OCD and suicidal ideation to avoid confusion with suicidal obsessions [10].

Table 2 summarizes some of the important studies that explain the risk of suicide in patients with OCD.

**Table 2:** Summary of some of the studies used to explain the risk of suicide in patients with OCD OCD: obsessive-compulsive disorder, SUD: substance use disorder, BD: bipolar disorder

Author name	Year of publication	Study Design	Sample size (if applicable)	Conclusions
Albert et al. [15]	2019	Systematic Review	N/A	Anxiety disorders, BD, SUD, past history of suicide, and hopelessness are predictors of higher suicide risk in OCD.
Kim et al. [16]	2016	Self Report	81	Perfectionism and alexithymia predict a higher risk of suicide in OCD.
Velloso et al. [17]	2016	Cross-sectional	548	Sexual, religious dimensions and past history of suicide attempt, positive family history of suicide are associated with a high risk of suicide in patients with OCD.
Fernández de la Cruz et al. [6]	2017	Case-cohort	36788	A previous suicide attempt predicts a high risk of suicide. Poisoning was the most common method used for suicide. Personality disorders and SUD increase suicide risk in patients with OCD.
Rachamallu et al. [10]	2017	Case Report	N/A	Suicidal obsessions are different from suicidal ideation. Suicidal obsessions are undesirable, stressful, and intrusive thoughts.

Body Dysmorphic Disorder

People with BDD are overly preoccupied with one or multiple features in their body which they believe look flawed. They repetitively and compulsively check the perceived defect in front of a mirror or ask others about their appearance. The preoccupation is obsessive and consumes many hours per day. Studies of twins showed that approximately 43% of genetic factors contribute to BDD symptoms. Bullying, childhood abuse, peer teasing are risk factors for the development of BDD [18]. Patients with BDD are often presented with anxiety and depression so their symptoms are commonly misdiagnosed as social phobia, MDD, or OCD [19]. Eating disorders like bulimia and anorexia nervosa are common disorders in patients with BDD [20]. BDD is present in about 25% to 39% of patients diagnosed with anorexia nervosa. Those patients have a high rate of hospitalization and a three times increase in suicide attempts [21]. Muscle dysmorphia is a form of BDD that is common among men. Men with muscle dysmorphia believe they are not muscular enough when in fact they might look unusually muscular. They may take anabolic steroids, follow a strict diet, and adhere to time-consuming work-out schedules. They might avoid social events and occupational activities because of the shame on their perceived appearance. Men with muscle dysmorphia have a poorer quality of life, an increase in substance use and suicide risk [22].

People with BDD often feel depressed. Studies found 24% to 28% of individuals diagnosed with BDD attempt suicide. Suicidal ideation and attempts are associated with an increase in symptom severity and marked psychosocial dysfunction. Risk factors for suicide ideation and attempts in BDD include comorbid BD, SUD, MDD, post-traumatic stress disorder, personality disorders especially borderline personality disorder [23]. An observational study (n = 200) found 94.6% of adolescents with BDD have reported distress and severe impairment socially, academically, and psychologically and 44.4% had suicide attempts [24].

Comorbid OCD and BDD

In the DSM-V, BDD was added to the obsessive-compulsive and related disorders category together with OCD, hoarding disorder, trichotillomania, and excoriation disorder. Both OCD and BDD share common features such as:

1. Genetics and environmental influences: past studies found high rates of both disorders among first degree relatives of patients with either OCD or BDD. They also found a high prevalence of other mental health disorders such as social phobia, depression, and eating disorders in these families [25]. A genetic study (n = 2,148) of adult twins found the common traits in OCD and BDD are due to genetic overlap of 64% in these two disorders [26]. Regarding the environmental factors, a cross-sectional study (n =100) found significantly higher rates of emotional and sexual abuse in BDD samples as compared to OCD samples. Both groups reported the same rates of physical abuse [26].

2. Socio-demographic profile: both disorders have very similar gender ratios and close social profiles with the exception of BDD which is slightly more common among single patients [27].

3. Psychopathological symptoms: people with BDD spend long hours performing ritualistic behaviors such as spending hours grooming, checking, and obsessing about their appearance. These repetitive behaviors are similar to compulsions in patients with OCD. People with BDD have less insight about their disorder and are more likely to be delusional compared to those with OCD [25].

 4. Cognitive characteristics: individuals with BDD have difficulties in accurately identifying facial emotional expressions. They are more likely to identify expressions as anger compared to OCD or normal individuals [28]. Both BDD and OCD patients are preoccupied with body symmetrical appearance [25,29].

5. Psychiatric and personality comorbidities: social phobia, MDD, SUD, suicidal ideation are common in both disorders; but are more prevalent in patients with BDD. Patients with BDD have lifetime suicidal attempt rates of 22% compared to 8% in patients with OCD. People with OCD are more likely to have obsessive-compulsive personality disorder while those with BDD are more likely to have narcissistic, histrionic, and avoidant personality disorders [25].

**Figure 1:**
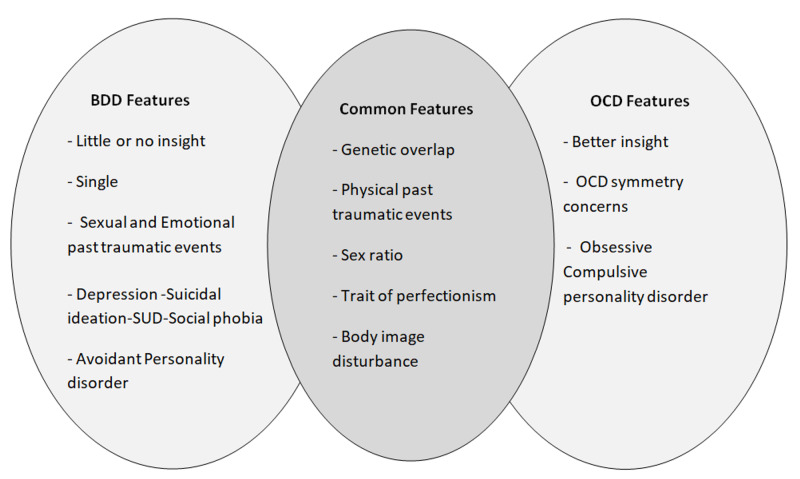
The differences and the common features between OCD and BDD OCD: obsessive-compulsive disorder, BDD: body dysmorphic disorder, SUD: substance use disorder

Figure 1 explains the differences and the common features between OCD and BDD [25].

Studies have found the prevalence of BDD in patients with OCD in a large patient sample was 8.7% to 15% compared to 3% in non-OCD. Similarly, the prevalence of OCD in patients with BDD was 37% to 16.7% compared to 8% in non-BDD patients [25]. A cross-sectional study (n = 137) compared three groups of patients with BDD, OCD, and comorbid OCD-BDD. The study found patients with comorbid OCD-BDD have higher rates of bulimia, SUD, Bipolar II disorder, and social phobia [30]. A cross-sectional study (n = 295) found people with comorbid OCD-BDD have high morbidity and poor psychosocial functions [31]. They also have higher rates of anxiety, schizotypal features, very poor insight, and suicidal ideation compared to those with BDD or OCD alone. The higher rates of comorbidities in the OCD-BDD group were the same as in the BDD group after controlling the symptom severity in BDD; however, it remained significant when compared with the OCD group [31]. The risk of comorbidity of OCD-BDD is three times higher in samples with a primary diagnosis of BDD compared to those with a primary diagnosis of OCD with 27.5% and 10.4%, respectively [25].

Study limitation

Our study is based on reviewing research articles published after 1997 and does not include possible important contributions from studies published prior to that. A systematic review in our study was not performed and no quality assessment of the selected research studies was done.

## Conclusions

OCD is a common psychiatric disorder associated with a high rate of psychiatric comorbidities and an increased risk of suicide. BDD is an underdiagnosed mental disorder that is commonly associated with depression, suicidal ideation, and attempts. The suicide risk in OCD is increased by the increase in the intensity of the obsessions, trait perfectionism and alexithymia. Individuals with BDD have poor insight and are usually single with past emotional and sexual traumatic events. The suicide risk in BDD is increased by the presence of comorbid SUD, MDD, eating disorders and personality disorders. OCD and BDD share common genetic and environmental risk factors. They also share common clinical features and sociodemographic profiles. Both OCD and BDD are related disorders that commonly coexist. People with comorbid OCD and BDD have a poor psychosocial outcome and are at high risk to develop social phobia, BD, SUD, and suicidal ideation. The suicide risk in OCD and BDD is mostly underestimated. Clinicians should be more aware of the various clinical presentations of BDD. It is recommended that dermatologists and cosmetic surgeons consult with mental health professionals for efficient diagnosis and proper referrals of patients with BDD. Patients with OCD should be properly screened for comorbid anxiety, depression, BDD, and suicidal ideation. Further original research studies are needed to investigate the relationship between OCD and BDD.
